# The Uninvited “Kiss”: When the Hunter Becomes the Hunted

**DOI:** 10.4269/ajtmh.18-0909

**Published:** 2019-03

**Authors:** Norman L. Beatty, Patricia L. Dorn, Stephen A. Klotz

**Affiliations:** 1 Division of Infectious Diseases, Department of Medicine, University of Arizona College of Medicine, Tucson, Arizona;; 2 Department of Biological Sciences, Loyola University New Orleans, New Orleans, Louisiana

The night was going great! Our collection efforts had been a success, but trapping kissing bugs is not easy. It takes a lot work. Our study site that night was in a remote canyon in southern Arizona, close to the border with Mexico. This small town with a rich mining history was once a booming community but now had less than 5,000 people. The homes are simple, many sitting off the ground, so-called pier and beam construction. Guided by our headlamps, we lugged our trapping equipment to a secluded home located against the canyon wall. I was already tired, having completed my infectious diseases clinic earlier in the day and then driving 2 hours down a number of winding mountain roads to reach the site. When I had arrived, some of our research team and collaborators were already settling in with the homeowners.

The night air was calm and surprisingly humid. A single street lamp was illuminating the natural stone road that had led me to a simple abode with a big problem. When you are bitten by a kissing bug, it can be a rather traumatic event: Suddenly waking up in the middle of the night with an itchy, painful welt, and a “creature” scurrying away in your bed. Can you imagine? However, this is a typical description of what most people experience when they contact us. Some 10% of bite victims may have anaphylaxis. Not to mention the bugs carry a parasite that can invade your heart. The home we were studying was infested with the largest kissing bug found in North America, *Triatoma recurva*. This unique triatomine is not usually associated with human dwellings, but it appears they have domiciliated many of the homes in this small town.

After setting up our traps and surveying the home, we sat down with the family in the cool summer night. A large picnic table set the stage for our interview: A place I could envision this family enjoying a meal or having a good laugh. You could tell they were close. Our conversation takes off with stories of these “monsters” day in and day out, preying on this humble family. They could not wait to bring us their “collection.” I held up an empty medicine bottle (an antihypertensive medication noted on the label), with a two-and-half-centimeter female *T. recurva*. This bug was big and alive! Her conenose was evident as I peered through the orange-tinted plastic. Taken aback by the size of this thing, I could not picture it crawling on me. Our discussions continued despite the shower of insects raining down on us as if we were being hit by beads being thrown during a Mardi Gras parade. Looking out at the ultraviolet lights that were illuminating the canyon, it reminded me of a small runway for a regional airport. The funny thing is we were not directing single engine airplanes to the landing strip but rather enticing blood-sucking insects to join the party. How exciting!

The comprehensive home and bite survey is an important element of our study, and these residents were eager to answer our questions. Bitten nearly on a nightly basis, they were now considering sleeping under mosquito nets. Our team wanted to learn more about the factors related to this unique interaction. Keeping an eye on the nearest illuminating sheet, my eyes were constantly scanning for the outline of a conenose. With hours upon hours of nighttime collecting under my belt, I now had some confidence in being able to spot them quickly. It reminded me of a game my son and I would play when out in the desert. I would often take him with me when collecting triatomines in the summer night, watching and waiting for the next conenose to land on our sheets. With childlike enthusiasm, we would try to outdo the other to see who can spot them the fastest.

Ten more minutes, maybe one more will show up? This is what I told myself when I knew things had slowed down. Oddly, it is as if a switch is turned off, and they cease to exist, at least on my illuminated sheets. We wrapped up the night and said our goodbyes. We would be seeing them in a couple days to complete the last part of the project, screening for Chagas disease. We loaded most of our gear into the bed of my truck and I took off. The adrenaline had now worn off and I needed some rest. I pulled into the driveway of the hotel, which was more like individual small cabins, divided into two. A door on each side with a welcome mat. Being that it was so late, I rang the bell to the manager’s quarters. “Well I didn’t know if you were going to show,” she exclaimed. It was now past 11. She walked me to my room and opened the door, fumbling to find the key. I turned on the lights and glanced around. The decor and furnishings were nice. She showed me where I could find the freshly washed linens and towels. “The hot water can be a bit finicky,” she said as she demonstrated how to use the shower. “Don’t forget, breakfast in the morning, 8 am, sharp,” she said and she was off, back to the comfort of her cabin. My intentions were to shower up and get some rest, but my exhaustion overtook me. I put down my pack and plopped right onto the bed. Lying on my back, my eyelids almost automatically closing. Taking some deep breaths, I could feel the desire to sleep taking hold. I remember opening my eyes and peeking at the door. The patio light was visible underneath but I thought nothing of it.

I just laid there. The bed was so comfortable. My mind was battling the natural course of the sleep–wake cycle. It is somewhat funny when you are fighting sleep; our minds tend to dance with the thoughts of the day. I distinctly remember thinking, “What if I was being hunted?” I mean, I am staying in the same canyon where I was collecting these kissing bugs. Why would they not be around? The door obviously had a huge crack in the jamb; they could walk right in. My eyes closed as my thoughts raced. I told myself, “I am fine, there is nothing to worry about,” as I faded away.

Suddenly, a thump. What was that? Is this really happening? My heart rate instantaneously soared but my eyes remained closed. No way is this happening. I am freaking myself out! Am I hearing things? Wait, was that a thump or a thud… that I felt, it was vibratory. Was something on the bed? Big enough for me to have felt it or did I hear it? Which one was it? Scrambling to understand what I just felt or heard, I was obviously confused. My senses were off, but how could I have imagined that? I opened my eyes. The fan blades above me were slowly rotating, the bedroom lights still on. Lying motionless, I turned my head to the left where I perceived this mysterious thump, my heart pounding as if I had just finished a marathon.

To my horror, it was real, and unimaginable. We looked at each other. I was now staring at a “creature” that was not science fiction. It wanted to suck my blood. An eerie feeling came over me. I am being hunted! She was an adult *T. recurva* with six thin long legs. A true bug. Her antennae slowly moving, probing the mattress sheet approximately two feet from my left leg, a conenose with a blood-sucking harpoon (proboscis) tucked underneath. Her intentions were simple, but her size was likely her downfall. Hard to land quietly with those awkward wings! Knowing what I was dealing with, I did not want her to get away. I slipped off the side of the bed, but surprisingly she just stayed in position. Maybe she felt like I was not a threat.

I grabbed a plastic cup out of the bathroom, slammed it down on top of her, and slid a piece of paper underneath. Now she was upset, flying around inside the cup. My hands clinched together on both ends. I could feel her moving about. There is no way I am sleeping in here tonight! I placed the critter into alcohol (because every kissing bug hunter has some alcohol in a conical tube close by) and jetted out of there as if a swarm of bees has entered the room. I slept in my truck that night. My own “collection” of triatomines resting in the back seat, albeit soaking in alcohol. I woke up to the early morning sun ironically without a welt in sight!

